# Decreased 1,25-Dihydroxyvitamin D3 level is involved in the pathogenesis of Vogt-Koyanagi-Harada (VKH) disease

**Published:** 2011-03-09

**Authors:** Xianglong Yi, Peizeng Yang, Min Sun, Yan Yang, Fuzhen Li

**Affiliations:** 1 Zhongshan Ophthalmic Center, Sun Yat-sen University, Guangzhou, Guangdong, China; 2 The First Affiliated Hospital of Chongqing Medical University, Chongqing Key Laboratory of Ophthalmology, Chongqing Eye Institute, Chongqing, China

## Abstract

**Purpose:**

1,25-Dihydroxyvitamin D_3_ [1,25(OH)_2_D_3_] has recently been found to be involved in the development of autoimmune diseases. This study was to investigate the expression and potential role of 1,25(OH)_2_D_3_ in the pathogenesis of Vogt-Koyanagi-Harada (VKH) disease.

**Methods:**

Blood samples were obtained from VKH patients and healthy individuals. Serum 1,25(OH)_2_D_3_ levels were measured using ELISA. Peripheral blood mononuclear cells (PBMCs) or cluster of differentiation (CD) 4^+^ T cells were cultured with or without 1,25(OH)_2_D_3_ in the presence of anti-CD3 and anti-CD28 for the measurement of cell proliferation and cytokines. The cell proliferation was detected using the Cell Counting Kit. The levels of interleukin (IL)-17 and interferon (IFN)-γ levels in the supernatants of PBMCs or CD4^+^ T cells were detected by ELISA.

**Results:**

1,25(OH)_2_D_3_ was significantly decreased in the serum of active VKH patients as compared with inactive VKH patients and controls. It significantly inhibited PBMCs proliferation and CD4^+^ T cell proliferation. It was also able to significantly inhibit the production of IL-17 and IFN-γ by both PBMCs and CD4^+^ T cells from VKH patients and controls.

**Conclusions:**

These findings suggest that decreased expression of 1,25(OH)_2_D_3_ may be involved in the development of VKH disease. 1,25(OH)_2_D_3_ may be potentially used in the treatment of this disease.

## Introduction

Vitamin D is produced in the skin upon exposure to sunlight or obtained from the diet [1-3]. Its receptor has been found in the immune cells and parts of these cells are able to produce Vitamin D3 [4-7]. 1,25(OH)_2_D_3_, the biologically active metabolite of Vitamin D3, has recently been shown to have immunomodulation property beside its involvement in the bone and calcium metabolism [8,9]. Recent studies showed that 1,25(OH)_2_D_3_ was able to skew the T cell compartment into a more anti-inflammatory and regulated state, as evidenced by inhibition of Th1 and Th17 cells and promotion of Th2 and T reg cells [10,11]. In Vitro experiments showed that 1,25(OH)_2_D_3_ could promote both innate and adaptive immune responses. 1,25(OH)_2_D_3_ receptor gene knockout mice showed a significantly higher susceptibility to several autoimmune disease models, such as experimental autoimmune encephalomyelitis [12,13], experimental autoimmune uveitis [14], and experimental allergic asthma [15]. Additionally, serum level of 1,25(OH)_2_D_3_ was found to be decreased in several human autoimmune diseases, such as multiple sclerosis [16-18], rheumatoid arthritis [19,20], Behçet's disease [21], Graves disease [22], and systemic lupus erythematosus [23,24]. All these results suggest that 1,25(OH)_2_D_3_ is a negative factor and that down-regulated expression of 1,25(OH)_2_D_3_ is possibly involved in the pathogenesis of these diseases.

Vogt-Koyanagi-Harada (VKH) disease is an autoimmune disease characterized by a bilateral granulomatous panuveitis and systemic disorders. It frequently results in severely decreased vision or even to blindness if not treated appropriately [25-27]. It is one of the most common uveitis entities in China as well as in the Far East of Asia [26,28-30]. Studies have showed that both enhanced T helper (Th) 1 and Th17 cell responses are involved in the pathogenesis of this disease.

In this study we examined the expression of 1,25(OH)_2_D_3_ and its possible involvement in the increased Th1 and Th17 response as already reported previously. Our results showed that a down-regulated expression of 1,25(OH)_2_D_3_ was present in active VKH patients and was possibly one of factors responsible for the increased Th1 and Th17 response in this disease.

## Methods

### Patients and controls

A total 25 adult patients with VKH disease (13 men and 12 women) with an average age of 38.4 years and a total 16 healthy individuals (9 men and 7 women) with an average age of 40.5 years were included in the study. All patients were diagnosed as having definite VKH disease according to the diagnostic criteria revised for VKH disease in an international committee on nomenclature [31]. Fifteen patients showed active intraocular inflammation (active uveitis stage), as evidenced by mutton fat keratic precipitates, cells in the anterior chamber and sunset glow fundus. The systemic findings included headache (54%), poliosis and alopecia (72%), vitiligo (23%), and tinnitus (46%). Ten active patients were at their first attack with disease duration <6 months, and 5 active patients showed recurrent intraocular inflammation with the duration of <2 years. Ten inactive VKH patients showed typical bilateral sunset glow fundus but without any intraocular inflammation (inactive uveitis stage), with the disease duration from 1 to 3 years. No systemic corticosteroids or other immunosuppressive agents were used at least for 2 weeks before being referred to our hospital and blood sampling in active VKH patients. Ten patients without active intraocular inflammation for at least 3 months after the treatment of corticosteroid and immunosuppressant were chosen as controls. No patients had received any dietary supplements before study entry. Blood samples were obtained from these active or inactive patients during November 1, 2009 to March 31, 2010, and the daily sun exposure time was estimated to be <1.5 h for all subjects. This study was approved by the Ethics Committee of the First Affiliated Hospital of Chongqing Medical University, and complied with the tenets of the Declaration of Helsinki.

### 1,25(OH)_2_D_3_ assays

Blood samples were collected from 8 active VKH patients, 7 inactive VKH patients and 8 healthy controls. Serum was obtained by centrifugation at 3,000× g for 10 min and stored at −70 °C until analysis. Serum 1,25(OH)_2_D_3_ level was measured using a commercially available ELISA kit (Immunodiagnostik, Bensheim, Germany) according to manufacturer’s instructions. An assay sensitivity level was 6.0 pg/ml.

### Cell isolation and culture

Anticoagulated blood samples were obtained from 11 active VKH patients, 7 inactive VKH patients and 12 healthy controls. peripheral blood mononuclear cells (PBMCs) were isolated using Ficoll-Hypaque density gradient centrifugation. Peripheral cluster of differentiation (CD) 4^+^ T cells were purified by human (h) CD4 microbeads according to the manufacturer’s instructions (Miltenyi Biotec, Palo Alto, CA). Cells were resuspended at 1×10^6^ cells/ml in medium RPMI 1640 (Gibco, Invitrogen, Carlsbad, CA) containing L-glutamine (2 mM), penicillin/streptomycin (100 U/ml), and 10% fetal calf serum. Cells were cultured with or without 1,25(OH)_2_D_3_ (Sigma-Aldrich, St louis, MO) in 37 °C, 100% humidity, 5% CO_2_ for measurement of cell proliferation and cytokines.

### Proliferation assay

For proliferation assay, PBMCs and CD4^+^ T cells suspension was transferred to a 96-well plate (200 μl/well), treated with or without 1,25(OH)_2_D_3_ (1×10^−7^ mol/l) and cultured for 5 days. Proliferation was measured using the Cell Counting Kit (CCK)-8 (Sigma-Aldrich, St louis, MO) assay, which is based on the conversion of water-soluble tetrazolium salt, WST-8 [2-(2-methoxy-4-nitrophenyl) −3-(4-nitrophenyl)-5-(2,4-disulfophenyl) −2H-tetrazolium, monosodium salt] to a water-soluble formazan dye upon reduction in the presence of an electron carrier by dehydrogenases [32]. The culture was performed in a 96-well plate in 200 μl of medium containing 20 μl CCK-8 for 3h at 37 °C. The OD was read at 450 nm using a multi-plate reader (SpectraMax M2^e^; Molecular Devices, Sunnyvale, CA).

### Cytokine analysis

For determination of interleukin (IL)-17 and interferon (IFN)-γ production, PBMCs and CD4^+^ T cells were stimulated with or without anti-CD3 (monoclonal antibody, 5μg/ml) and anti-CD28 antibodies (1μg/ml; eBioscience, San Diego, CA) for 72 h at a concentration of 1×10^6^ cells/ml. Supernatants were collected and the levels of IL-17 and IFN-γ were measured using the human IL-17 DuoSet ELISA development kit and human IFN-γ DuoSet ELISA development kit (R&D Systems, Minneapolis, MN) with a detection limit of 15.6 pg/ml.

### Statistics

One-way ANOVA, paired-sample *t*-test were applied using SPSS 12.0. Data were expressed as mean±SD. A level of p<0.05 was considered to be statistically significant.

## Results

### 1,25(OH)_2_D_3_ expression in the serum of VKH patients and controls

**Figure 1:**
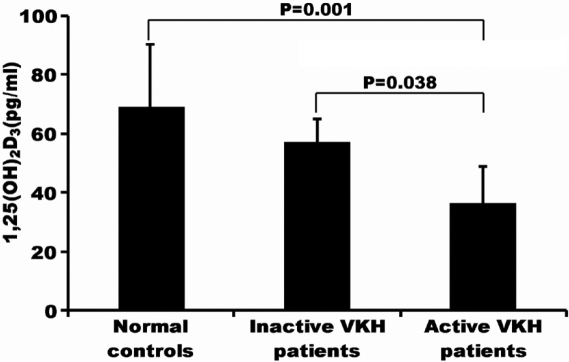
1,25(OH)2D3 in the serum from active VHK patients (n=8), inactive VHK patients (n=7) and normal controls (n=8).

1,25(OH)_2_D_3_ could be detected in the serum from both VKH patients and controls. The level of 1,25(OH)_2_D_3_ in active VKH patients (36.3±12.7pg/ml) was significantly lower than that in inactive VKH patients (57.3±8.00 pg/ml, p=0.038) and normal controls (69.1±21.21 pg/ml, p=0.001; Figure 1). There was no significant difference between inactive VKH patients and controls concerning the serum level of 1,25(OH)_2_D_3_.

### Effect of 1,25(OH)_2_D_3_ on proliferation of PBMCs and CD4^+^ T cells

**Figure 2:**
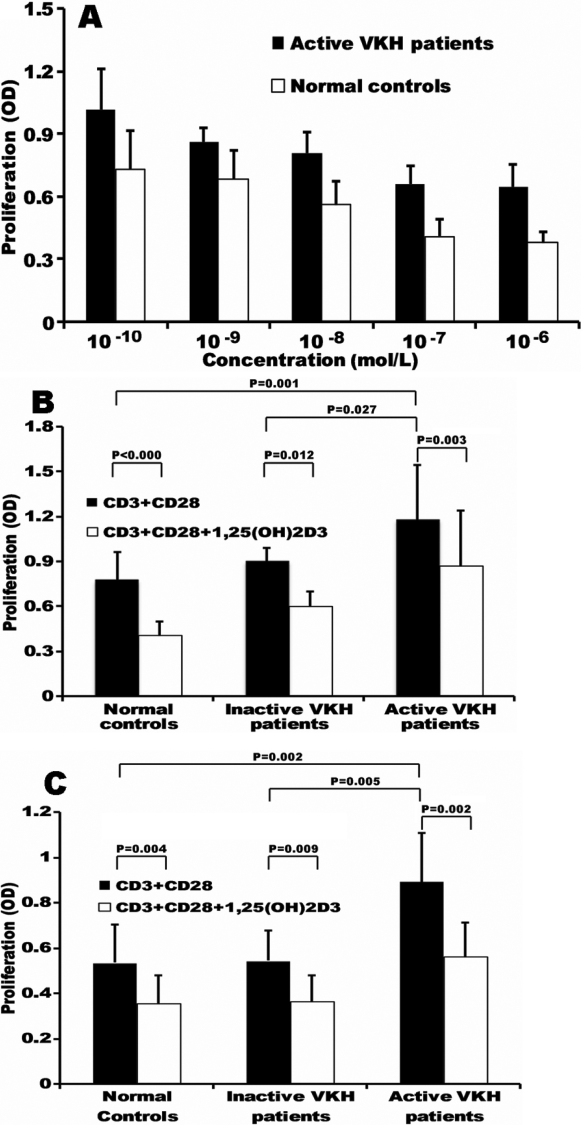
Effect of 1,25(OH)2D3 on the cell proliferation of PBMCs and CD4+ T cells from VKH patients and controls. **A**: Cell proliferation of PBMCs from active VKH patients (n=6) and normal controls (n=6) stimulated with 1,25(OH)2D3 of different concentrations. **B**: Cell proliferation of PBMCs from active VHK patients (n=11), inactive VHK patients (n=7) and normal controls (n=12) stimulated with or without 1,25(OH)2D3 (1×10^−7^mol/l). **C**: Cell proliferations of CD4+ T cells from active VHK patients (n=7), inactive VHK patients (n=7) and normal controls (n=9) stimulated with or without 1,25(OH)2D3 (1×10^−7^mol/l).

A primary experiment with different concentrations of 1,25(OH)_2_D_3_ showed that it significantly inhibited the proliferation of PBMCs from active VKH patients and controls. Furthermore, the result showed that its inhibitory effect was in a dose-dependent manner (Figure 2B). As 1×10^−7^ mol/l of 1,25(OH)_2_D_3_ could induce a significant suppression on the proliferation of PBMCs, this concentration was used in the following experiments. Both PBMCs and CD4^+^ T cells from active VKH patients showed a significantly higher cell proliferation as compared with the inactive VKH patients and controls. 1,25(OH)_2_D_3_ significantly inhibited the cell proliferation of PBMCs and CD4^+^ T cells from all VKH patients and controls (Figure 2C). There was no difference among these tested three groups concerning the inhibitory percentage.

### Effect of 1,25(OH)_2_D_3_ on the production of IL-17

**Figure 3:**
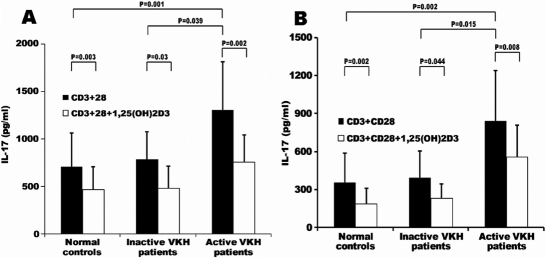
IL-17 production by PBMCs and CD4+ T cells. Cells were cultured with anti-CD3 and anti-CD28 antibodies in the presence or absence of 1,25(OH)2D3 for 72 h. **A**: IL-17 production by PBMCs from active VHK patients (n=11), inactive VHK patients (n=7) and normal controls (n=12). **B**: IL-17 production by CD4+ T cells from active VHK patients (n=7), inactive VHK patients (n=7) and normal controls (n=9).

PBMCs and CD4^+^ T cells from VKH patients and controls cultured with 1,25(OH)_2_D_3_ in the presence of anti-CD3 and anti-CD28 antibodies were used to evaluate the influence of this molecule on IL-17 production. A significantly increased production of IL-17 was observed in active VKH patients as compared with inactive VKH patients (PBMCs: p=0.039; CD4^+^ T cells: p=0.015) and controls (PBMCs: p=0.001; CD4^+^ T cells: p=0.002). 1,25(OH)_2_D_3_ significantly inhibited the production of IL-17 by PBMCs and CD4^+^ T cells from the VKH patients and controls (Figure 3A,B). There was no difference among the tested three groups concerning the inhibitory percentage.

### Effect of 1,25(OH)_2_D_3_ on the production of IFN-γ

**Figure 4:**
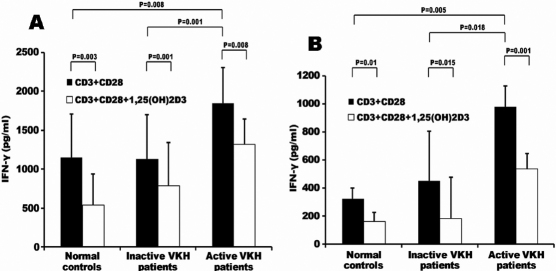
IFN-γ production by PBMCs and CD4+ T cells. Cells were cultured with anti-CD3 and anti-CD28 antibodies in the presence or absence of 1,25(OH)2D3 for 72 h. **A**: IFN-γ production by PBMCs from active VHK patients (n=11), inactive VHK patients (n=7) and normal controls (n=12). **B**: IFN-γ production by CD4+ T cells from active VHK patients (n=7), inactive VHK patients (n=7) and normal controls (n=9).

PBMCs and CD4^+^ T cells from VKH patients and controls cultured with 1,25(OH)_2_D_3_ in the presence of anti-CD3 and anti-CD28 antibodies were used to examine the influence of this molecule on the production of IFN-γ. IFN-γ production by both PBMCs and CD4^+^ T was significantly higher in active VKH patients as compared with inactive VKH patients (PBMCs: p=0.001, CD4^+^ T cells: p=0.018) and normal controls (PBMCs: p=0.008, CD4^+^ T cells: p=0.005). 1,25(OH)_2_D_3_ significantly down-regulated the production of IFN-γ by PBMCs and CD4^+^ T cells from VKH patients and controls (Figure 4A,B). Similar to the IL-17 result, no difference was found concerning the inhibitory percentage of 1,25(OH)_2_D_3_ on the production of IFN-γ by PBMCs and CD4^+^ T cells among the tested three groups.

## Discussion

In this study, we showed that serum level of 1,25(OH)_2_D_3_ was significantly decreased in the serum of active VKH patients. 1,25(OH)_2_D_3_ was able to inhibit cell proliferation of PBMCs and CD4^+^ T cells. It also down-regulated the IL-17 and IFN-γ production by activated PBMCs and CD4^+^ T cells from both VKH patients and controls. These results suggested that the decreased expression of 1,25(OH)_2_D_3_ may be involved in the development of VKH disease.

Vitamin D is a member of the steroid thyroid superfamily of nuclear receptors. The immune regulatory effects observed in other autoimmune diseases stimulated us to investigate whether 1,25(OH)_2_D_3_ was involved in the pathogenesis of VKH disease. For this purpose, we first detected the 1,25(OH)_2_D_3_ level in the serum of VKH patients. Our results showed that the serum level of 1,25(OH)_2_D_3_ was significantly decreased in active VKH patients. This result is generally consistent with those reported in other autoimmune diseases, such as multiple sclerosis [16,17], rheumatoid arthritis [19], and systemic lupus erythematosus [23,24]. This result suggest that downregulated expression of 1,25(OH)_2_D_3_ was associated with the active intraocular inflammation in VKH patients.

The decreased expression of 1,25(OH)_2_D_3_ in active VKH diseases, but not in inactive VKH patients, suggests that it may be involved in the pathogenesis of the intraocular inflammation in this disease. As T cell proliferation is one of important factors in the immune response or in the inflammation, we further investigated the effect of 1,25(OH)_2_D_3_ on cell proliferation. Our results showed that 1,25(OH)_2_D_3_ significantly inhibited the cell proliferation of PBMCs and CD4^+^ T cells from all VKH patients and controls. These results suggest that PBMCs and CD4^+^ T cells from VKH patients and controls are similar in the sensitivity to it with respect to cell proliferation. Our result is consistent with early report, in which 1,25(OH)_2_D_3_ was found to suppress cell proliferation of PBMCs or CD4^+^ cells in both human and animal models [19,24,33].

As IL-17 is found to have an important role in autoimmune diseases including VKH disease, we further examined the effect of 1,25(OH)_2_D_3_ on the secretion of IL-17. Consistent with our previous study [34], a significantly increased production of IL-17 was observed in active VKH patients. 1,25(OH)_2_D_3_ significantly inhibited the production of IL-17 by PBMCs and CD4^+^ T cells from VKH patients and controls. These results demonstrated an inhibitory effect of 1,25(OH)_2_D_3_ on IL-17 production reported in other autoimmune diseases [19,24,33]. Unexpectedly, we failed to find any difference regarding the inhibitory percentage of 1,25(OH)_2_D_3_ on the IL-17 production among the tested three groups, suggesting that the sensitivity of PBMCs and CD4^+^ T cells to 1,25(OH)_2_D_3_ is not different concerning IL-17 production.

Upregulated Th1 response has been found as one of the mechanisms of autoimmune diseases including VKH disease [34-36]. Our study further examined whether 1,25(OH)_2_D_3_ could influence the expression of IFN-γ, a typical cytokine of Th1 cells, in VKH patients. The result showed a significantly increased IFN-γ production by PBMCs and CD4^+^ T cells in active VKH patients. This result is consistent with the results reported previously [34]. Our study also showed that 1,25(OH)_2_D_3_ was able to inhibit the production of IFN-γ by PBMCs and CD4^+^ T cells from both VKH patients and controls. Therefore, the sensitivity of both PBMCs and CD4^+^ T cell to 1,25(OH)_2_D_3_ were not different among the tested three groups concerning IFN-γ production.

In summary, our study revealed that decreased 1,25(OH)_2_D_3_ level was associated with active intraocular inflammation in VKH patients. 1,25(OH)_2_D_3_ could significantly inhibit cell proliferation of PBMCs and CD4^+^ T cells, and down-regulated expression of IL-17 and IFN-γ. These results suggest that down-regulated expression of 1,25(OH)_2_D_3_ may be one of mechanisms for the increased IL-17 and IFN-γ in the development of VKH disease. Our study also suggests that upregulating 1,25(OH)_2_D_3_ might provide a new strategy for the treatment of VKH disease.
